# Improved Cardiovascular Risk among Hispanic Border Participants of the Mi Corazón Mi Comunidad Promotores De Salud Model: The HEART II Cohort Intervention Study 2009–2013

**DOI:** 10.3389/fpubh.2015.00149

**Published:** 2015-06-03

**Authors:** Hendrik Dirk de Heer, Hector G. Balcazar, Sherrie Wise, Alisha H. Redelfs, E. Lee Rosenthal, Maria O. Duarte

**Affiliations:** ^1^Department of Physical Therapy and Athletic Training, Northern Arizona University, Flagstaff, AZ, USA; ^2^School of Public Health, University of Texas Health Science Center, Houston, TX, USA; ^3^Project on Community Health Worker Policy and Practice, Institute for Health Policy, University of Texas School of Public Health, El Paso, TX, USA; ^4^Department of Public Health Sciences, The University of Texas at El Paso, El Paso, TX, USA

**Keywords:** community resources, parks and recreation, community health workers, U.S.–Mexico border, Hispanic, cardiovascular disease, cohort

## Abstract

**Background:**

Community resources (parks, recreational facilities) provide opportunities for health promotion, but little is known about how to promote utilization of these resources and their impact on cardiovascular disease risk (CVD).

**Methods:**

This cohort study evaluated the impact of an intervention called Mi Corazon Mi Comunidad (MiCMiC), which consisted of promoting use of community physical activity and nutrition resources by Promotoras de Salud/Community Health Workers. Participants were assessed at baseline and following the 4-month intervention. Attendance records were objectively collected to assess utilization of intervention programing.

**Results:**

A total of five consecutive cohorts were recruited between 2009 and 2013. Participants were mostly females (86.0%), on average 46.6 years old, and 81% were low in acculturation. Participants who completed follow-up (*n* = 413) showed significant improvements in reported health behaviors and body composition. Higher attendance significantly predicted greater improvements. The baseline to 4-month *change* for the *highest* vs. the *lowest* attendance quartiles were for weight (−5.2 vs. +0.01 lbs, *p* < 0.001), waist circumference (−1.20 vs. −0.56 inches, *p* = 0.047), hip circumference (−1.13 vs. −0.41 inches, *p* < 0.001); hours of exercise/week (+3.87 vs. +0.81 hours, *p* < 0.001), proportion of participants eating five servings of fruits and vegetables/day (+54.7 vs. 14.7%, *p* < 0.001).

**Conclusion:**

Following the Promotora-led MiCMiC intervention, substantial improvements in health behaviors and modest improvements in cardiovascular risk factors were found. Greater utilization of community resources was associated with more favorable changes. This study provided preliminary evidence for the effectiveness of Promotora-led interventions for promoting use of existing community resources in CVD risk reduction.

## Introduction

From 2000 to 2010, the Mexican-American population in the U.S. increased rapidly from 20.6 million to 31.8 million and in 2010 represented an estimated 63% of the total U.S. Hispanic population (1). More than 85% of the Mexican-American population is geographically concentrated in the West and South. The four U.S.–Mexico Border States (New Mexico, Texas, California, and Arizona) have the highest proportion of residents of Hispanic descent at more than 30% of the total population (1). Addressing the health care needs of the Hispanic border population presents unique challenges, given the bilingual, bi-national environment, the limited resources of the population (2), and the high risk for cardiovascular and metabolic conditions (3–5). To address cardiovascular risk factors among Mexican-American border residents, several programs have been developed such as the health promotion curriculum called Salud Para Su Corazon (SPSC; for the health of your heart) (6–8). SPSC is aimed at Mexican-Americans and utilizes Community Health Workers/’Promotores de Salud’ (9, 10) to function as links between health care providers and community members for outreach and curriculum delivery.

Recently, ecological approaches that take into account the environment have been suggested for common chronic disease prevention and control (11). Evidence supporting the impact of environmental factors on health behaviors and outcomes has strengthened this approach. For example, it has been found that proximity to and access to recreational facilities and grocery stores impacts physical activity and nutrition behaviors and consequently cardiovascular and metabolic risk (12, 13). To date, however, few evidence-based projects have prospectively evaluated the extent to which community interventions can promote utilization of community resources, and whether increased resource utilization is associated with beneficial cardiovascular disease risk (CVD) outcomes.

To this end, the Health Education Awareness Research Team (HEART) Phase II cohort study was implemented in a large metropolitan U.S.–Mexico border area from 2009 to 2013 (14, 15). In partnership with the local parks and recreations department and Promotores de Salud employed by a local clinic, five cohorts of high-risk Mexican-American residents were recruited with the aim of promoting utilization of existing community resources and evaluate CVD risk outcomes. It was found that Promotores were able to successfully facilitate using the community resources, as evidenced by an average attendance of over 21 physical activity and nutrition sessions over a 4-month period (Balcazar et al., under review). Using the HEART Phase II data, the current paper describes the behavioral and CVD outcomes of the study. The primary hypothesis that was tested for the current paper was that increased utilization of community resources, as measured by attendance of a larger number of health sessions, was associated with a greater improvement in cardiovascular risk factors as well as dietary and physical activity behaviors.

## Materials and Methods

### Study setting

The study was conducted in El Paso, TX, USA, located directly at the U.S.–Mexico border. Approximately 80% of residents in the area are Mexican-American (16). Educational attainment and median income are lower than state and national averages, and the proportion of residents without health insurance is higher (16). The intervention was implemented in two zip codes, selected by a community leadership council formed in HEART Phase I (17, 18). These zip codes were in the lower valley area, which is characterized by a greater proportion of Mexican-American residents (almost 95%), a lower socio-economic status and less access to health care compared to the rest of El Paso (19). In prior research, residents of these zip codes have been found to have a very high prevalence of CVD risk factors (17, 20).

### Study design and programing

The HEART Phase II study was a cohort study with two measurement times: at baseline and 4-month follow-up. The decision to not build a randomized trial was guided by the HEART Community Health Advisory and Leadership Council, which oversaw the implementation of HEART Phase I (17, 18). From 2009 to 2013, a total of 753 Mexican-American border residents participated across 5 cohorts, 413 of whom completed all the 4-month follow-up measures. These 413 participants are the focus of the current analyses.

The programing for the intervention was called *‘Mi Corazon Mi Comunidad’* (MiCMiC; My Heart My Community), which integrated best practice methods from the CDC Task Force on Community Preventive Services (21, 22). MiCMiC programing focused on facilitating access to resources that can promote heart-healthy behaviors available in the community. This consisted of increasing access to existing facilities, and promoting participation in organized activities led by Promotoras de Salud. Three partners in the community supported the implementation of the MiCMiC intervention. These partners included: the YWCA, the city’s Parks and Recreation department, and Centro San Vicente, a community health clinic located in the study area. Three Promotoras de Salud were hired to guide the implementation of the program.

Activities focused on physical activity, dietary intake, and heart-healthy education. For physical activity, the focus was on facilitating use of parks and recreational facilities (the YWCA) through a free membership to the YWCA for the duration of the study and organized activities such as walking groups and dance classes. For dietary behaviors, nutrition-related activities such as cooking classes, grocery store tours, and coffee talks were led by the Promotoras. The Su Corazon Su Vida (SCSV) curriculum (8), a comprehensive Promotora outreach program aimed at promoting heart-healthy behaviors and tailored toward Mexican-American populations, was also taught by the Promotoras to facilitate the promotion of heart-healthy behaviors.

For each of the five study cohorts, a 4-month calendar of activities was developed by the research team. This calendar outlined weekly activities including YWCA activity classes, activity sessions in the parks (i.e., walking groups), SCSV curriculum classes, and other nutrition activities (i.e., grocery store tours, cooking demos). The weekly calendar typically consisted of between four and five structured activities per week including at least one nutrition-focused activity, two physical fitness-focused activities, and one SCSV class or family activity. Structured sessions were held both at the YWCA and at the parks. In addition, participants were encouraged to engage in other unstructured activities such as using the YWCA during non-class hours or engaging in activities in the parks at any time.

Attendance goals were developed by the Promotoras de Salud in collaboration with the research team and community partners. The total goal that was agreed upon was to aim for a total of 28 sessions for each participant over the 16-week period. To record utilization of community resources, total attendance was recorded for all activity and nutrition sessions. At the YWCA (where Promotores de Salud were located during the study period), a record-keeping system (using an electronic card system) tracked use of the facilities. Attendance was also recorded by the Promotores de Salud for the other study activities (SCSV classes, activity sessions in the parks, grocery store tours, etc.) (15).

Across all cohorts, on average, 21.6 total sessions were attended during the 4-month period, with 5 of 6 (83.6%) of participants attending at least one session, and 75% attending at least 3 sessions (Balcazar et al., under review). Small incentives (water bottles, t-shirts, cooking materials) were provided to facilitate continued program attendance.

### Participants and recruitment

**Figure 1:**
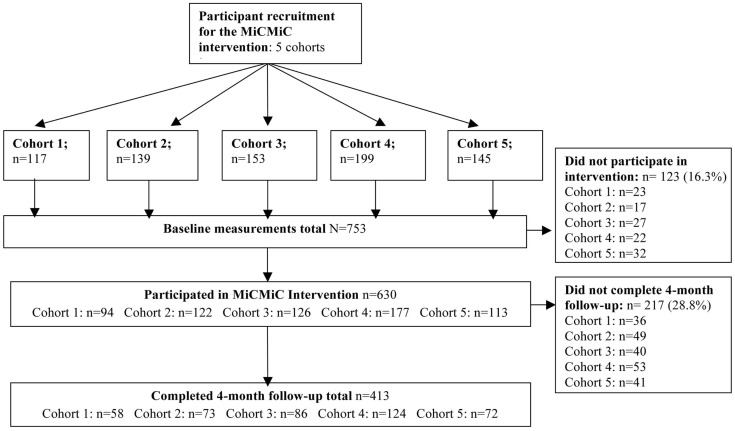
**Flowchart of participants in the HEART Phase II community-based cohort study on the U.S.–Mexico border from 2009 to 2013**.

Participants for the current study were recruited through snowball sampling, recruitment flyers, and door-to-door knocking. Participants were recruited by Promotores de Salud at community health fairs, the YWCA, recreation centers, community health clinics, through personal contacts, and through Spanish speaking radio and TV programing (15). A list of interested eligible participants was developed by community health workers from these recruitment activities. No records were kept to distinguish how many participants were approached and recruited through each technique. Inclusion criteria were that participants were residents of the two study zip codes, they were adults (18 and over), not pregnant, of Hispanic descent, and able to participate in all MiCMiC activities. Five subsequent cohorts were recruited. The intervention was implemented from 2009 through 2013. Figure 1 describes participants and recruitment. All procedures were approved by the Institutional Review Boards of the University of Texas Health Science Center at Houston and the University of Texas at El Paso.

Although 83.6% of participants attended at least one session, complete data were only available on 16-week follow-up from 413 of the 753 participants. The proportion of participants completing follow-up did improve with every cohort from cohort 1 through cohort 4, but still remained below expectations. Although the numbers are comparable to other similar studies [e.g., Ref. (24)], the lack of completion of follow-up may have been related to the structure of the project incentives, which emphasized attendance over completion of follow-up measures. Participants who completed follow-up were not different on any of the indicators of insurance, income, education, employment status, and gender from participants who did not complete follow-up (data not shown). However, they were about 2 years older (*p* < 0.001), and were slightly lower in indicators of acculturation, as 83.1% were low on the SASH acculturation scale vs. 75.9% of those who did not complete follow-up, *p* = 0.017.

### Measures

At baseline and 4 months, participants completed clinical measurements including height, weight, waist circumference, hip circumference, and blood pressure (the average of the last two of three measurements was taken). Measures were completed following standard procedures (i.e., American Heart Association). To make it easier to translate and disseminate the data to the participants and the community, a sum score of CVD risk factors including screening practices, presence of chronic conditions, and health behaviors was calculated (0–11, with 1 assigned for each of the unhealthy options). Items included (1) recent screening for diabetes, and (2) high cholesterol (not screening counted as 1, screening a 0); baseline measurements of (3) BMI (overweight or not), and (4) waist circumference (abdominal obesity or not), (5) high blood pressure, (6) reported diabetes, (7) reported high cholesterol; and behaviors including (8) physical activity at least 30 min five times/week, (9) smoking, (10) consumption of five daily fruits and vegetables, and (11) whether participants had attended a class to improve their health in the past 6 months.

A survey (available in Spanish and English) included items about demographic characteristics (age, sex, gender, socio-economic status) and a series of questions assessing health habits. Bilingual interviewers administered the survey. Acculturation was assessed with the SASH acculturation questionnaire (23). Fruit and vegetable consumption was assessed through asking participants if they ate five servings of fruits/vegetables per day. For physical activity, participants were asked if they exercised 30 min/day three times per week, and the number of hours per week they engaged in exercise. Finally, using items from the 2008 National Survey on Drug Use and Health (25), participants were asked if they currently smoked cigarettes and if they had smoked in the past 30 days.

### Analyses

All primary analyses were conducted using SPSS version 21.0 (SPSS Inc., Chicago, IL, USA). Descriptive statistics and frequency distributions were used to describe demographic characteristics. Regression analyses using Generalized Estimating Equations (GEE) to take into account the “nested” data structure (participants within cohorts) were used to compare baseline to 4-month comparisons. Finally, we tested whether a larger number of total sessions attended were associated with a greater change in health outcomes. Attendance was categorized into quartiles, and it was tested whether changes in health outcomes from baseline to follow-up were significantly different between each quartile. In all regression models, covariates included sex, age, gender, acculturation, and socio-economic status.

## Results

### Demographic characteristics and baseline health status

Participants who completed follow-up (*n* = 413) were mostly female (86% were female) and had an average age of 46.6 years (SD = 12.8). Socio-economic status was low, as about 70% had an annual household income under $20,000, and almost half did not have health insurance. More than 80% of participants were low in acculturation, the majority were born in Mexico (65.9%), and about five out of six participants (86.0%) chose Spanish as their preferred interview language. At baseline, 75% of participants had abdominal obesity and almost 65% had hypertension or pre-hypertension (see Table 1).

**Table 1:** **Clinical characteristics at baseline and 4-month follow-up of 413 Mexican-American participants in the HEART Phase II community-based cohort study on the U.S.–Mexico border from 2009 to 2013**.

Variable	Baseline	4-Month follow-up	*p*-Value^a^
Height (inches)	63.11 (3.16)	N/A	
Weight (pounds)	178.17 (38.00)	175.97 (37.52)	<0.001**
BMI (mean kg/m^2^)	31.39 (6.19)	31.07 (6.20)	<0.001**
Underweight	0.2%	0.5%	<0.001**
Healthy weight	13.9%	14.7%	
Overweight	32.0%	32.3%	
Obese	53.9%	52.5%	
Waist (inches)	38.72 (5.27)	37.80 (5.50)	<0.001**
Abdominal obesity males (40″)	55.0%	50.8%	0.494
Females (>35″)	75.3%	67.7%	0.004**
Hip circumference (inches)	44.14 (5.14)	43.41 (5.11)	<0.001**
Systolic BP (mm Hg)	126.87 (18.90)	126.19 (16.76)	0.535
Diastolic BP (mm Hg)	76.70 (9.52)	75.64 (9.25)	0.257
Any pre-hypertension or hypertension	63.7%	63.3%	0.943
CVD risk sum score (of 11 factors)	5.60 (1.78)	3.74 (1.66)	<0.001**
Health behaviors
Exercise 30 min 3×/week (% yes)	43.2%	85.2%	<0.001**
Hours per week	2.51 (3.85)	5.44 (3.20)	<0.001**
Eat five fruits/vegetables/day (% yes)	35.8%	66.1%	<0.001**
Do you currently smoke cigarettes	8.4%	6.8%	0.002**

^a^*p-*Values based on linear (for continuous variables), logistic (for dichotomous variables), or ordinal probit regression analyses (for categorical variables) using generalized estimating equations (GEE) controlling for cohort membership. These analyses included a predictor variable “time,” which was included in the model to test whether the value at time 1 was significantly different from the value at time 2. *Significant difference on *p* < 0.05, **significantly different on *p* < 0.01.

### Change from baseline to follow-up

Findings for all 413 participants from baseline to 4-month follow-up are presented in Table 1. Self-reported health behaviors showed significant improvements. Whereas at baseline, just over 40% of participants reported exercising three times a week for 30 min, at follow-up this was five out of six participants (*p* < 0.001) and average hours per week of reported exercise increased by almost 3 hours (from 2.51 to 5.44 hours, *p* < 0.001). Intake of at least five daily servings of fruits and vegetables increased from 33.3% at baseline to 67.4% at follow-up (*p* < 0.001). Smoking declined from 8.4 to 6.8% (*p* = 0.002).

For clinical indicators, on average for all participants, weight (2 lbs), waist circumference (1″), and hip circumference (0.75″) all significantly decreased (*p-*values <0.001). About 5% fewer participants were categorized as abdominally obese. Systolic and diastolic blood pressure were slightly lower at post-test (about 1 mm Hg), but these changes were not significant. Finally, the CVD risk sum score was reduced by about two points from 5.6 risk factors at baseline to and average of 3.7 risk factors at follow-up (*p* < 0.001).

### Number of sessions attended and changes in health outcomes

Finally, we tested whether greater participation in the MiCMiC intervention was associated with greater improvements in behavior and CVD. Greater participation was defined as greater attendance at the health programing. Participants were grouped into quartiles by median attendance, which was 0 sessions for participants in the lowest quartile, a median of 6 sessions for quartile 2, a median of 22 sessions for participants in quartile 3, and a median of 55 sessions for participants in the highest quartile of attendance over the 4-month period. Quartile 1 was used as the reference group, and it was tested whether improvements in outcomes among participants in quartiles 2, 3, and 4 were significantly different from quartile 1.

It was found that greater attendance was significantly associated with improvements in body composition (see Table 2). Compared to quartile 1 (0.01 lbs increase), quartiles 3 (2.5 lbs reduction), and 4 (5.2 lbs reduction) showed significantly greater weight reduction (*p* < 0.001) at 4-month follow-up. For waist and hip circumference, findings were similar. Compared to quartile 1 (0.56″ waist reduction), participants in quartiles 4 (1.58″ reduction, *p* = 0.008) and 3 (1.20″ reduction, *p* = 0.047) had significantly greater reductions in waist circumference at 4-month follow-up. For hip circumference compared to quartile 1 (0.41″ reduction), quartile 4 had a significantly greater reduction (1.13″ reduction, *p* = 0.001). Greater attendance was not associated with changes in blood pressure.

**Table 2:** **Clinical changes of Mexican-American participants in the HEART Phase II community-based cohort study on the U.S.–Mexico border from 2009 to 2013, grouped by quartiles of intervention attendance**.

Variable	Weight (pounds)				Hip circumference (inches)				Waist Circumference (inches)			
	Pre	Post	Change	*p*-Value^a^	Pre	Post	Change	*p*-Value	Pre	Post	Change	*p*-Value
**TOTAL ATTENDANCE**												
Quartile 1 (reference)	184.55	184.56	+0.01		44.11	43.70	−0.41		39.68	39.10	−0.56	
Quartile 2	179.92	178.76	−1.16	0.121	44.37	43.94	−0.43	0.850	39.04	38.25	−0.79	0.491
Quartile 3	168.80	166.35	−2.45	<0.001**	43.45	42.78	−0.67	0.344	38.07	36.49	−1.58	0.008**
Quartile 4	179.76	174.61	−5.15	<0.001**	44.39	43.26	−1.13	<0.001**	38.83	37.63	−1.20	0.047*

	**Hours of exercise per week**				**Exercise 30 min 3 days per week (% yes)**				**Eat five fruits/vegetables per day (% yes)**			
	**Pre**	**Post**	**Change**	***p-*Value**	**Pre**	**Post**	**Change**	***p*-Value**	**Pre**	**Post**	**Change**	***p*-Value**

**TOTAL ATTENDANCE**												
Quartile 1 (reference)	2.48	3.29	+0.81		38.0 %	65.3 %	+27.3%		31.6%	46.3%	+14.7%	
Quartile 2	2.49	4.03	+1.54	0.174	42.6 %	82.4 %	+39.8%	0.002**	33.3%	69.4%	+36.1%	<0.001**
Quartile 3	1.91	4.75	+2.84	0.003**	43.1 %	94.1 %	+51.0%	<0.001**	32.0%	68.9%	+36.9%	0.008**
Quartile 4	2.76	6.63	+3.87	<0.001**	48.5 %	99.0 %	+51.5%	<0.001**	35.5%	80.2%	+54.7%	<0.001**

^a^*p*-Values are based on regression analyses using generalized estimating equations (GEE) taking into account nested data structure, controlling for: baseline value, age, sex, income, and acculturation. To assess whether greater attendance is associated with greater improvements in health outcomes changes are compared across quartiles of attendance (Quartile Preference). *Significant compared to reference group on *p* < 0.05, **significant on *p* < 0.01.

Greater attendance was further consistently associated with greater improvements in health behaviors related to dietary intake and physical activity (see Table 2). Participants in quartile 1 increased their hours of exercise per week by 48 min/week (0.81 h), whereas participants in quartile 4 increased almost 4 h (*p* < 0.001 compared to quartile 1). The proportion of participants in quartile 1 who reported eating five fruits and vegetables daily increased by 14.7% (from 31.6 to 46.3%) compared to 54.7% increase in quartile 4 (from 35.5 to 80.2%; *p* < 0.001 for comparison).

## Discussion

The current study evaluated whether participation in an intervention aimed at facilitating use of community resources resulted in changes in health behavior and CVD risk. The study was implemented among Mexican-American residents of the U.S.–Mexico border area, who were mostly low in socio-economic status and acculturation. Promotores de Salud were employed to facilitate use of community resources, which included parks and YWCA recreational facilities. Findings indicate that participants improved their health behaviors substantially from baseline to follow-up, including improvements in hours per week of exercise and reported nutrition intake. Statistically significant, but clinically small improvements in indicators of body composition were found, including weight, BMI, waist, and hip circumference. Greater improvements were found among participants who attended more intervention sessions. These findings provide support for the effectiveness of Promotora-led interventions aimed at facilitating access to community resources among high-risk underserved Mexican-American border residents.

Our findings confirmed that the study population was at very high risk for CVD. For example, 85% were overweight, 75% had abdominal obesity, and almost 65% had pre-hypertension or hypertension. The population was also low in socio-economic status and acculturation. These demographics are characteristic of the U.S.–Mexico border population and illustrate the need and opportunity for interventions tailored to these populations such as the MiCMiC program.

As a result of the MiCMiC intervention, substantial improvements were found in behavioral outcomes including reported physical activity and dietary intake. For example, two out of five participants reported exercising three times a week for 30 min at baseline, which improved to over four of five participants at follow-up. The increase in number of people attending health promotion related activities per week was further confirmed by objectively collected attendance data as described in greater detail elsewhere (Balcazar et al., under review). Changes were also very large between the highest and lowest attendance quartiles. Participants in the highest quartile increased their hours of weekly activity by about 3 h more compared to participants in the lowest quartile. Also, despite having a similar baseline proportions of people meeting recommendations for fruit and vegetable intake (about one-third), 80% of participants in the highest quartile of attendance met fruit and vegetable intake at follow-up compared to just over 45% for the lowest attendance quartile.

Although the effects found for reported health behaviors and objectively collected attendance records were large, changes in clinical indicators were small. Therefore, although statistically significant, the magnitude of some of the clinical changes (for example, a 5-lb weight difference between the lowest and highest quartiles) is limited in terms of CVD. It is possible that the follow-up time was too short to detect changes large enough to be of clinical relevance. The magnitude of the changes is similar or greater, however, to prior similar studies. For example, Ayala (24) reported the findings of a Promotora model aimed at increasing physical activity by facilitating use of parks and free exercise classes. The study found reductions in waist circumference at 6 months of 0.35 cm, which increased to 2 cm at 12 months, and a weight reduction of 1 lb at 6 months and just over 2 lbs at 12 months (resulting in a 0.3 kg/m^2^ BMI reduction). These numbers are similar or slightly lower than the numbers found in our study, despite the much shorter study period at 4 months. In addition, the current study expanded on the prior research by Ayala (24) in showing that greater utilization was associated with greater improvements.

The findings of the current study were similar to several other studies utilizing Promotora-led health promotion interventions such as Camina por Salud [*Walks for Health* (26)], Pasos Adelante [*Steps Forward* (27)], and Familias Sanas y Activas [*Healthy and Active Families* (24)]. Although these studies showed that Promotoras were able to increase access to care through, for example, facilitating immunizations (9) and health behavior change (24, 26, 27), little research to date had evaluated whether Promotoras can facilitate utilization of existing recreational facilities. The current study contributes to this literature by demonstrating that a Promotora-led program increased use of community resources related to heart-healthy behaviors among high-risk Mexican-American border residents with limited access to care. This study expands the previous research by showing that greater utilization predicted greater changes in self-reported behaviors and body composition, and that the findings of the current study were repeatable across five consecutive cohorts.

The findings of this study are further consistent with ecological perspectives (11, 15), which have reported that environmental restructuring, through, for example, enabling access to community resources and recreational facilities, is an essential part of widespread health promotion. The findings further demonstrate that integration of a model where Promotores de Salud shows potential in facilitating access to health resources (17, 18, 24, 26–28). This model may be particularly relevant in areas whose residents are underserved, have limited access to care and are at high risk for CVD such as the U.S.–Mexico border.

### Limitations

The current study has several limitations. First, the number of participants who was measured at 4-months was relatively low compared to the number of original study participants, despite 83.6% attending at least one session, and 75% attending three or more sessions. The lack of completion of follow-up may have been due to an incentive structure that emphasized attendance over completion of follow-up. As an illustration, 33% of participants who were in the highest quartile of attendance did not complete the follow-up, suggesting that participants may have focused on session attendance over completion of the follow-up. In addition, anecdotal reports indicated that participants thought that the amount of time it took to complete all the measurements (2+ h) was prohibitive to their participation in the follow-up. Second, there was a selection bias for the cohort as the participants self-selected into the intervention. As a result, several characteristics of the sample are not representative of the area (i.e., over 80% were female and only 21.6% were employed full time). Other characteristics, however, such as the low acculturation, income, educational attainment, and limited access to care, are representative of the population in the area. Further, although the majority of Mexican-Americans in the U.S. live in proximity to the border, the unique nature of the area may limit the ability to generalize the results of the current study to other locations. Also, while some of the findings regarding clinical outcomes (particularly anthropometric data) were statistically significant, the effects were too small to have a large clinical relevance at the 4-month follow-up. In addition, many of the health behaviors were self-reported, and social desirability in answers to reported behaviors has to be taken into account. Finally, the YWCA was only provided for the duration of the study, and the authors have no knowledge about continued attendance at the YWCA after the study ended.

## Conclusion

The current study demonstrates that a community health worker approach can be utilized to facilitate utilization of community resources among a cohort of high-risk Mexican-American border residents. Increased utilization of community resources was associated with modest, but significant improvements in indicators of body composition and substantial improvements in health behaviors over a 4-month period. The findings further included five consecutive cohorts, suggesting that the study may be repeatable, at least in the current setting. Further research with stronger experimental control, longer follow-up times, and additional objective cardiovascular risk factors is needed to evaluate whether facilitating access to community resources can provide sustainable health improvements among high-risk Hispanic border residents.

## Conflict of Interest Statement

The authors declare that the research was conducted in the absence of any commercial or financial relationships that could be construed as a potential conflict of interest.
